# A Case-Based Look at Healthy Weight Loss for Survivors of Cancer

**Published:** 2016-03-01

**Authors:** Kristy K. Hager

**Affiliations:** Wellmont Cancer Institute, Kingsport, Tennessee

## CASE STUDY

Ms. D is a 47-year-old female who was diagnosed with stage IIIA triple-negative breast cancer in 2014. She completed neoadjuvant therapy with doxorubicin, cyclophosphamide, and paclitaxel and then had a left mastectomy and radiation therapy. 

During an appointment in the survivorship clinic, she reveals to her advanced practitioner (AP) and dietitian that she is concerned about her weight. Her body mass index (BMI) is 42.5 kg/m², which places her in the obese category. She has multiple questions regarding weight loss, including the use of phentermine, and is interested in losing weight "the fastest way possible." She is only eating one meal a day in an attempt to lose weight. Other than obesity, her physical examination is normal, and her laboratory results are unremarkable.

The AP discussed Ms. D’s BMI with her. She reviewed the risk of cancer recurrence, noting that a BMI of ≥ 35 kg/m² may be associated with a higher risk of recurrence. She emphasized the role of exercise in weight loss. She encouraged Ms. D to begin an exercise routine at 5 minutes per day and work up to a goal of 30 minutes of moderate to strenuous exercise at least 5 days per week. Ms. D stated that there is a track near her home and that she could begin walking daily there.

The AP stressed the importance of following good nutritional guidelines and eating from every food group. She reviewed the safety data on phentermine, specifically the cardioexcitatory effects. She noted that long-term efficacy and safety evidence is lacking.

The dietitian discussed Ms. D’s desire for rapid weight loss. She educated her on healthy weight loss, which may seem slow but is much better for the body. She encouraged colorful, healthy food from all food groups such as fruits and vegetables, whole grains, beans, lean meats, and avoiding processed items such as fast foods and convenience foods. The dietitian discussed the importance of hydration, and Ms. D revealed that she drinks about three cola drinks and at least one glass of sweetened iced tea per day. The dietitian told her that caffeine-free and calorie-free beverages are best for hydration, stressing that water is the healthiest choice.

The dietitian asked about Ms. D’s meal setting. She said that she eats in front of the TV daily. It was recommended that she eliminate distractions and eat with the TV off, so she would be more cognizant of portion size and stop eating when she was satisfied. She encouraged Ms. D to take smaller bites and chew slowly to help minimize overeating by giving the satiety center in the brain time to receive messages of fullness from the stomach.

The dietitian discussed the need for weight loss utilizing adequate nutritional intake vs. a "quick-fix" pill such as phentermine. A calorie-restricted diet is still required for weight loss when using phentermine. Weight is usually rapidly regained after the drug is stopped unless there are behavioral and lifestyle changes. She gave Ms. D a tip sheet for successful weight loss, including having consistent meal times, avoiding skipping meals, using portion control, limiting alcohol intake, eating meals in one specified location, and adequate hydrating with noncaloric, caffeine-free liquids. They brainstormed about healthy snacks to have on hand, and Ms. D said she likes apples, raisins, and yogurt. The dietitian gave her some ideas about planning ahead for lunches to take to work, so she could avoid fast food. They discussed other times of temptation to overeat and ways to prevent it, all with a goal of long-term, sustainable weight reduction.

## ARTICLE

Overweight and obesity are terms used for ranges of weight that are greater than what is generally considered healthy for a given height (Centers for Disease Control and Prevention, 2012). As many as 84,000 cancer diagnoses each year are attributed to obesity, and obesity is implicated in 15% to 20% of total cancer-related mortality (National Cancer Institute, 2012; Calle, Rodriguez, Walker-Thurmond, & Thun, 2003; American Cancer Society, 2012).

Defined as a condition characterized by the excessive accumulation and storage of fat in the body, obesity can be an unwelcome part of cancer survivorship. Obesity has been associated with an increased risk for recurrence of colorectal and breast cancers (in postmenopausal women; Marian, 2013). Obese men seem to be at increased risk of developing biologically aggressive prostate cancer (Efstathiou et al., 2007). A BMI of ≥ 35 kg/m² may be associated with an increased risk of colon cancer recurrence and mortality (Meyerhardt et al., 2003; Dignam et al., 2006) as well as other cancers. Oncology patients are often encouraged to prevent weight loss during treatments. However, when a patient completes active treatment and transitions into survivorship, weight loss may be needed.

After a cancer diagnosis and successful treatment, patients may be afraid and anxious about cancer recurrence. Obesity can place individuals at increased risk of developing second primary malignancies (Park, Choi, Jang, Jun, & Kang, 2010; Li, Daling, Porter, Tang, & Malone, 2009a; Li et al., 2009b), so patients are often told to achieve a healthy weight. Patients may be desperate for help with losing weight and often fall prey to "quick-fix" schemes. Due to the anxiety of the cancer diagnosis and the trauma of cancer treatment, patients usually desire fast weight loss, with less regard to the safety of the weight-loss method. The methods patients find readily available often promise huge amounts of weight loss with little to no effort or exercise and may be costly, unhealthy, and even dangerous.

Many cancer survivors report attempts to make healthy changes after a cancer diagnosis (Demark-Wahnefried, Aziz, Rowland, & Pinto, 2005). Careful planning is needed to help oncology survivors lose unwanted pounds. Ideally, a visit with a registered dietitian is part of survivorship care planning. This article will provide the AP with information about popular weight-loss methods and offer healthy weight-loss strategies to share with cancer survivors.

## POPULAR WEIGHT-LOSS METHODS

The overall amount of weight a person needs to lose in a certain situation can often be a daunting number to absorb. The degree of weight loss required may cause patients to believe that the quicker the weight comes off, the better off they will be. Using a Google search, a person can find an overwhelming number of weight-loss methods and the most recent advice related to achieving weight loss effortlessly. Some of the popular trends in use today include the extreme HCG (human chorionic gonadotropin) diet and phentermine pills. During this search, one would also find some outlandish claims such as the Twinkie diet, the tapeworm diet, the cotton-ball diet, and ear stapling.

It is important for patients to remember that successful weight loss must have two key components: (1) It must be gradual, and (2) it requires work. If a diet or product sounds too good to be true, it usually is (Academy of Nutrition and Dietetics, 2016). Patients may not understand these components, so it falls to the registered dietitian or the AP (in the absence of a registered dietitian) to provide education.

**HCG Diet**

The HCG diet is one of the most popular current diet methods. According to an article in the May 2013 issue of the *Annals of Pharmacotherapy*, the HCG diet originally gained popularity in the 1950s (Goodbar, Foushee, Eagerton, Haynes, & Johnson, 2013). Dr. Albert Simeons promoted HCG injections in combination with an ultralow-calorie diet of 500 calories per day. This article stated: "This weight loss strategy claims to redistribute body fat from the hips, thighs, and stomach, without undesirable effects such as hunger and irritability" (Goodbar et al., 2013).

*Dangers*: There are dangers associated with the use of higher amounts of HCG (Goodbar et al., 2013). In fact, Goodbar and colleagues reported an incident of deep vein thrombosis and bilateral pulmonary embolism associated with the initiation of the HCG diet. The HCG diet has "very few efficacy studies and no significant safety studies associated with its use" (Goodbar et al., 2013). Of the six studies identified for assessment of its efficacy, only one study was associated with significant weight reduction (Asher & Harper, 1973). In addition, overall findings from a review of the available literature about the effects of HCG in the treatment of obesity "failed to demonstrate fat redistribution, reduction in hunger, or an improvement in well-being" (Toffle, 2011). Goodbar and colleagues (2013) noted that as the popularity of the HCG diet continues to increase, so do the potential adverse events associated with the management of weight loss via an unproven strategy.

**Phentermine**

There is a growing public mindset that a person can just "pop a pill" and successful weight loss will occur. Successful, permanent weight loss is more than just the use of pills for appetite suppression. One example of a weight loss pill is phentermine. Phentermine is "a noradrenergic drug that stimulates noradrenaline release and reduces food intake by acting on â-adrenergic receptors in the perifornical hypothalamus" (Bray, 1993).

*Dangers*: Phentermine exhibits actions similar to amphetamines. There are major safety concerns due to the cardioexcitatory effects of phentermine as monotherapy (Hainer & Aldhoon-Hainerová, 2014). Although it was approved for short-term treatment of obesity by the US Food and Drug Administration (FDA) in 1959, long-term safety and efficacy evidence is still lacking (Li & Cheung, 2009).

**FDA-Approved Weight Loss Drugs**

In 2012, the FDA approved two long-term weight loss drugs: (1) a combination of the drugs phentermine and topiramate (PT) and (2) lorcaserin. The combination of PT is indicated for use as an adjunct to a reduced calorie diet and increased physical activity for chronic weight management in adults with an initial BMI of 30 kg/m² or greater (obese) or 27 kg/m² or greater (overweight) in the presence of at least one weight-related comorbidity such as hypertension, type 2 diabetes mellitus, or dyslipidemia (Vivus, 2014).

Lorcaserin is a serotonin 2C receptor agonist indicated as an adjunct to a reduced calorie diet and increased physical activity for chronic weight management in adults with an initial BMI of 30 kg/m² or greater (obese) or 27 kg/m² or greater (overweight) in the presence of at least one weight-related comorbid condition such as hypertension, dyslipidemia, or type 2 diabetes (Arena Pharmaceuticals, 2012). The mechanism of action is stimulation of satiety centers found throughout the brain, which is thought to reduce hunger by the production of opiomelanocortin neurons in the hypothalamus (Mahgerefteh, Vigue, Freestone, Silver, & Nguyen, 2013).

*Dangers*: Although data showed that PT did help patients lose weight over a 1-year time frame, there were also issues with harmful metabolic acidosis, memory, and attention or language problems (Woloshin & Schwartz, 2014). Common adverse effects reported with PT include paresthesia and dry mouth (Woloshin & Schwartz, 2014). According to the prescribing information, the most common adverse reactions are paresthesia, dizziness, dysgeusia, insomnia, constipation, and dry mouth (Vivus, 2014).

Lorcaserin was approved by the FDA based largely on three major phase III trials (Mahgerefteh et al., 2013). However, weight was regained once use of the medication was discontinued, and patient outcomes were no better than in patients receiving placebo (Mahgerefteh et al., 2013). According to the package insert, the most common adverse reactions reported with lorcaserin in nondiabetic patients are headache, dizziness, fatigue, nausea, dry mouth, and constipation (Arena Pharmaceuticals, 2012). The most common adverse reactions in diabetic patients are hypoglycemia, headache, back pain, cough, and fatigue (Arena Pharmaceuticals, 2012).

## CRITERIA FOR APPROVAL OF WEIGHT-LOSS MEDICATIONS

The FDA considers two criteria for approval of weight-loss medications: (1) whether participants lose 5% or more weight with the study drug vs. placebo, and (2) whether at least 35% of participants taking the drug lose at least 5% body weight (Woloshin & Schwartz, 2014). For PT, both of these FDA criteria were met Woloshin & Schwartz, 2014). However, the combination medication was not the only intervention. Trial participants were also counseled to increase exercise and reduce caloric intake while taking any medications provided. Results over an additional follow-up of 1 year also showed that participants regained about 25% of the weight that they had lost (Woloshin & Schwartz, 2014). Lorcaserin efficacy is also dependent upon decreasing caloric intake and increasing activity.

**Dietary Restrictions/Focus**

Sometimes patients resort to particular diet regimens that restrict part of or sometimes all of a specific food group in the quest for weight loss. Some examples include the cabbage soup diet, the grapefruit diet, and the no-carbohydrate diet. Adequate nutritional intake to meet nutritional needs should be balanced. Eating well is important to overall health, and when intake is not balanced or a particular item or group of food is eliminated, the nutritional result is unhealthy and inadequate. Balanced eating includes good protein sources (from plant or animal), fiber (including a variety of fruits and vegetables), carbohydrates, vitamins, minerals, healthful fats, and fluid (Academy of Nutrition and Dietetics, 2016).

## HEALTHY WEIGHT LOSS

The American Society of Clinical Oncology has developed several priorities to address the obesity and cancer link. These priorities include education, awareness, clinical guidance, tools, resources, research promotion, and policy/advocacy (Ligibel et al., 2014). Evidence shows the most effective interventions for weight loss combine nutritional education, diet and exercise counseling, and behavioral strategies to help obese patients acquire the skills they need to successfully change their eating habits and to become more physically active (Wu, Gao, Chen, & van Dam, 2009; Norris et al., 2004).

It is the position of the Academy of Nutrition and Dietetics that successful weight management to improve overall health requires a lifelong commitment to healthful lifestyle behaviors, emphasizing sustainable and enjoyable eating practices and daily physical activity (Seagle, Strain, Makris, Reeves, & American Dietetic Association, 2009). Ultralow-calorie diets do not meet the recommended daily allowances for health.

Using inappropriate weight-loss methods may lead to less-than-desirable results. When a person ceases such inappropriate calorie restrictions, which cannot be sustained over a lifetime, weight will often increase. Patients are not taught how to eat in a healthy way or about the importance of exercise when they are just "popping a pill" for weight loss. There is a general misconception about getting healthy as a "diet" vs. a "lifestyle change."

In the absence of a registered dietitian, the tips found in the Table can be applied by the AP and used as a guide to help patients in their weight-loss journey (Hager, 2014).

**Table 1:**
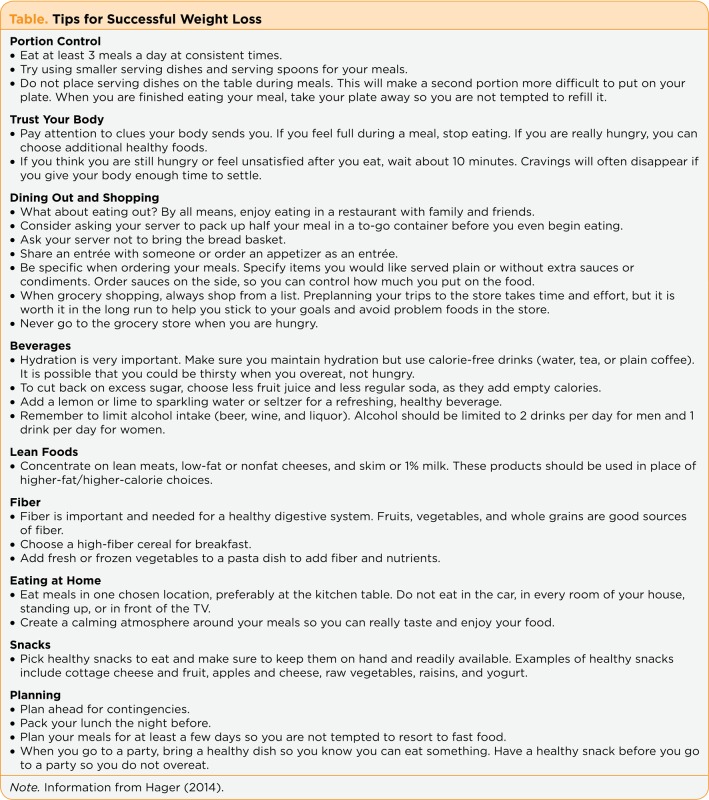
Tips for Successful Weight Loss

## SUMMARY

The AP plays a vital role in helping cancer survivors achieve and maintain a healthy weight. The AP must maintain an accurate knowledge base of the current diet trends and their safety. When considering weight-loss medications or alternative methods as part of a patient’s regimen, the AP must weigh both the risks and the benefits and effectively communicate them to the patient. Counseling, support, and referrals to a registered dietitian are all part of the oncology AP’s role.
